# *LEP* rs7799039 and *LEPR* rs1137101 gene variants are not associated with clinical features in patients with metabolic syndrome in the Turkish population

**DOI:** 10.1093/labmed/lmae061

**Published:** 2024-08-13

**Authors:** Marjan Jabbarli, Naci Senkal, Fatima Ceren Tuncel, Yasemin Oyaci, Merve Guzel Dirim, Murat Kose, Sacide Pehlivan, Alpay Medetalibeyoglu

**Affiliations:** Istanbul University, Istanbul Medical Faculty, Department of Internal Medicine, Istanbul, Turkey; Istanbul University, Istanbul Medical Faculty, Department of Internal Medicine, Istanbul, Turkey; Istanbul University, Institute of Graduate Studies in Health Sciences, Molecular Medicine, Istanbul, Turkey; Istanbul University, Istanbul Medical Faculty, Department of Medical Biology, Istanbul, Turkey; Istanbul University, Istanbul Medical Faculty, Department of Medical Biology, Istanbul, Turkey; Istanbul University, Istanbul Medical Faculty, Department of Internal Medicine, Istanbul, Turkey; Istanbul University, Istanbul Medical Faculty, Department of Internal Medicine, Istanbul, Turkey; Istanbul University, Istanbul Medical Faculty, Department of Medical Biology, Istanbul, Turkey; Istanbul University, Istanbul Medical Faculty, Department of Internal Medicine, Istanbul, Turkey; Northwestern University, Feinberg School of Medicine, Chicago, IL, US

**Keywords:** leptin, leptin receptor, metabolic syndrome

## Abstract

**Objectives:**

Genetic predisposition plays a role in the etiology of metabolic syndrome (MetS), an important health problem worldwide. Leptin (LEP), produced by adipose tissue, plays a crucial role in the development of MetS. In this study, we evaluated the effects of *LEP* and *LEP receptor (LEPR)* variants on clinical findings and risk of developing MetS in the Turkish population.

**Methods:**

A total of 320 patients were included in the study, of whom 150 were patients with MetS and 170 were healthy controls. DNA was extracted from blood samples. *LEP* rs7799039 and *LEPR* rs1137101 variants were genotyped using the polymerase chain reaction–based restriction fragment length polymorphism method. The genotype distributions of these variants and clinical and laboratory findings were compared.

**Results:**

The *LEP* rs7799039 GA and AA genotypes and A allele frequencies were higher in participants with MetS than in the control group. For *LEP* rs7799039, the genotype AA-GA was higher in males, and the GG genotype was higher in females. On analyzing the clinical outcomes associated with these variants, it was observed that individuals possessing *LEP* rs7799039 GA and AA genotypes displayed elevated levels of triglycerides. In addition, those with the AG-GG genotype of *LEPR* rs1137101 had lower mean hemoglobin levels.

**Conclusion:**

Our results showed that the *LEP* rs7799039 and *LEPR* rs1137101 variants may be associated with both the risk of MetS development and clinical findings. Among the various contributors to MetS, a genetic predisposition is commonly recognized as the primary cause.

## Introduction

Metabolic syndrome (MetS), an important health problem worldwide, is a cluster of conditions that increases the risk of developing cardiovascular disease, stroke, and type 2 diabetes (T2DM). It is characterized by a combination of factors including abdominal obesity, high blood pressure, high blood glucose levels, elevated triglycerides, and low levels of high-density lipoprotein (HDL) cholesterol.^1^ MetS affects approximately 20% to 25% of the adult population worldwide and occurs due to aging of the population, increasing life expectancy, obesity, sedentariness, and nutritional imbalance. Patients with MetS are 3 times more likely to have a stroke or heart attack and twice as likely to die from it than are healthy individuals. Furthermore, individuals with metabolic syndrome have 5 times the risk of developing T2DM compared with healthy individuals.^2^ Multiple genetic and environmental factors play roles in the development of MetS.^3^

Leptin (LEP) is a hormone produced by adipose tissue and plays a crucial role in regulating energy balance and body weight. LEP increases calorie expenditure and decreases adenosine triphosphate production and appetite.^4^ It regulates metabolism by binding to its receptor in the hypothalamus.^5^ The LEP receptor (LEPR) is a single-transmembrane protein belonging to a superfamily of cytokine receptors. These homodimers can activate Janus kinases to activate transcription.^6^ The *LEP* gene is located at position 7q31.3 and consists of 3 introns and 3 exons spanning approximately 18 kb.^7^ The *LEPR* gene on chromosome 1p31 encodes a single transmembrane protein 1165 amino acids in length and is distributed in many tissue types.^8^ Several single nucleotide variants (SNVs) have been identified in the highly polymorphic *LEP* and *LEPR* genes that are potentially associated with the pathophysiology of obesity, diabetes, and related complications. *LEP* (rs7799039, -2548 G>A) and *LEPR* (rs1137101, 668 A>G) variants have been reported to be associated with increased body mass index (BMI) in different ethnic populations.^9,10^

In terms of the connection between *LEP* and *LEPR* gene variants and obesity, there is limited information regarding the presence of MetS. Therefore, in this study, we aimed to evaluate the effect of the *LEP* rs7799039 and *LEPR* rs1137101 variants on the development of MetS in the Turkish population.

## Material and Methods

### Study Population

A total of 150 patients diagnosed with MetS (92 females, 58 males) attending the Department of Internal Medicine, Istanbul University, Istanbul Faculty of Medicine, were included in the study. Patients were diagnosed with MetS using the National Cholesterol Education Program Adult Treatment Panel III diagnostic criteria.^11^ According to this guideline, 3 criteria from waist circumference >102 cm in men or >88 cm in women, blood pressure ≥130/85 mmHg, fasting blood glucose ≥110 mg/dL (6.1 mmol/L), serum triglyceride ≥150 mg/dL (1.7 mmol/L), and HDL <40 mg/dL (1.03 mmol/L) in men or <50 mg/dL (1.29 mmol/L) in women are sufficient for the diagnosis of MetS. Individuals diagnosed with chronic infectious diseases, malignancies, or genetic abnormalities were excluded from the study. The control group consisted of 170 healthy individuals (87 females, 83 males) with normal blood glucose levels and no chronic disease.

BMI was calculated using the formula BMI = weight (kg) / (height [m^2^]). Laboratory results for various tests, including fasting blood glucose, hemoglobin A1c (HbA1c), C-peptide, insulin, creatinine, aspartate transaminase (AST), alanine transaminase (ALT), cholesterol, triglyceride, low-density lipoprotein (LDL), HDL, total cholesterol, C-reactive protein (CRP), thyroid function tests, creatinine, hemogram, complete urine test, and spot urine total protein/creatinine ratio, were collected and analyzed.

### Genotyping

**Figure 1.**
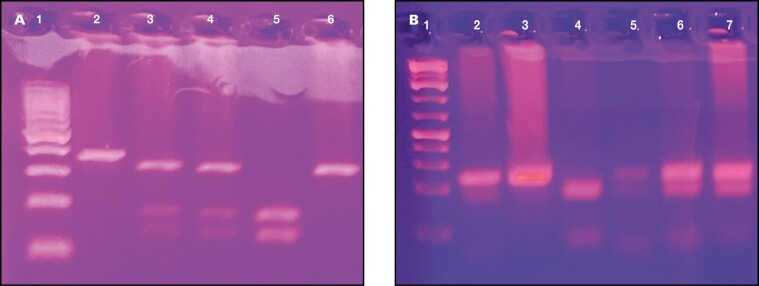
A: Representative gel image of *LEPR* gene rs1137101 variant. Lane 1:100 bp DNA ladder; Lane 2: nondigested polymerase chain reaction (PCR) product; Lane 3-4: AG genotype; Lane 5: GG genotype; Lane 6: AA genotype; B: Representative gel image of *LEP* gene rs7799039 variant. Lane 1: 100 bp DNA ladder; Lane 2: nondigested PCR product; Lane 3: GG genotype; Lane 4: AA genotype; Lane 5-6-7: GA genotype.

Three milliliters of peripheral blood samples from all participants were placed in EDTA tubes. DNA was isolated from blood samples using a commercial kit (Elk Biotech) according to the manufacturer’s instructions. *LEP* rs7799039 and *LEPR* rs1137101 were genotyped using the polymerase chain reaction–based restriction fragment length polymorphism method described previously.^10,12^ Primer sequences and binding temperatures used in the study are shown in TABLE 1. The HhaI restriction enzyme (NEB, New England Biolabs®) was used for *LEP* rs7799039 and the MspI (NEB) enzyme was used for *LEPR* rs1137101. Genotyping was performed by visualizing the samples after agarose gel electrophoresis (%2.5) under UV light. *LEP* rs7799039 genotypes were evaluated as GG: 242, GA: 242, 181, 61, and AA: 181, 61 bp. *LEPR* rs1137101 genotypes were AA: 416 bp, AG: 416, 229, 187 bp, and GG: 229, 187 bp. (FIGURE 1A and 1B)

**TABLE 1.** PCR Conditions for Each Polymorphism

	Primer sequences	Annealing temperature/ cycle
*LEP* rs7799039	F:5ʹ-TTTCCTGTAATTTTC CCGTGAG-3ʹ R:5ʹ-AAAGCAAAGACAGGCATA AAAA-3ʹ	53°C / 38 cycle
*LEPR* rs1137101	F: 5ʹ-GCCTAATCCAGTATTTTATATCTG-3ʹ R: 5ʹ-GCCACTCTTAATACCCCCAGTAC-3ʹ	60°C / 36 cycle

### Statistical Analysis

The evaluation of statistical data was conducted StataMP-64 software. The continuous variables were represented through the median value. The χ^2^ test was utilized to differentiate between groups, and the χ^2^ test and Fisher’s exact test were used to analyze the genotypes and allele distribution of *LEP* rs7799039 and *LEPR* rs1137101, respectively, between the case and control groups. The calculation of odds ratio (OR) and 95% CI was also performed. The *P* values were considered 2-tailed, and those with a value less than .05 were considered significant.

## Results

A total of 320 subjects, including 150 patients with MetS and 170 healthy controls, were genotyped for the *LEP* rs7799039 and *LEPR* rs1137101 polymorphisms. The genotype and allele distributions of *LEP* rs7799039 and *LEPR* rs1137101 in the patient and healthy control groups are shown in TABLE 2.

**TABLE 2.** Genotype Distribution and Allele Frequencies of *LEP* rs7799039 and *LEPR* rs1137101 in Patients and Controls

*LEP* rs7799039	Patients	Controls	OR Exp(B)	95% CI	*P*
Genotype	n = 150 (%)	n = 170 (%)			
GG	34 (22.7)	78 (45.9)	0.346^b^	0.212-0.563^b^	**.001** ^**b**^
GA	89 (59.3)	68 (40)	0.315^a^	0.188-0.631^a^	**.001** ^a^
AA	27 (18)	24 (14.1)	0.389^a^	0.196-0.774^a^	**.007** ^a^
Allele					
G	160 (53.3)	224 (65.9)			
A	140 (46.7)	116 (34.1)	1.690^b^	1.228-2.334^b^	**.002** ^**b**^
HWEp	0.02	0.15			
*LEPR* rs1137101					
Genotype					
AA	72 (48)	112 (65.9)	1.553^a^	0.747-3.231^a^	.239^a^
AG	60 (40)	41 (24.1)	0.707^a^	0.325-1.535^a^	.380^a^
GG	18 (12)	17 (10)	1.227^b^	0.608-2.478^b^	.594^b^
Allele					
A	204 (68)	265 (88)			
G	96 (32)	75 (22)	1.663^b^	1.168-2.366^b^	**.005** ^**b**^
HWEp	0.32	0.00			

HWEp, Hardy-Weinberg equilibrium in polyploids; OR, odds ratio

a OR (95%CI) was adjusted by age and sex, ^b^Fisher’s Exact Test. Statistically significant values are shown in bold.

### *LEP* rs7799039

The *LEP* rs7799039 genotype distribution was statistically different between subjects with MetS and controls. The *LEP* rs7799039 AA and GA genotypes were higher in patients than in healthy controls (*P* = .007, OR = 0.389 95%CI: 0.196-0.774; *P* = .001, OR = 0.315, 95%CI: 0.188-0.631, respectively). The GG genotype was more prevalent in healthy controls than in patients with MetS (*P* = .001, OR = 0.346, 95%CI: 0.212-0.563). Also, the A allele frequency was more prevalent in patients with MetS than in healthy controls (*P* = .002, OR = 1.690, 95%CI: 1.228-2.334).

### *LEPR* rs1137101

The prevalence of AA, AG, and GG of *LEPR* rs1137101 was 58%, 40%, and 12%, respectively, in patients and 65.9 %, 24.1 %, and 10 %, respectively, in healthy controls. There was no statistically significant difference in the *LEPR* rs1137101 genotype distribution between patients and healthy controls (*P* > .05). The G allele of *LEPR* rs1137101 was more frequent in patients with MetS than in healthy controls (*P* = .005, OR=1.663, 95%CI: 1.168-2.366).

In the control group, a deviation from the Hardy-Weinberg equilibrium for the *LEPR* rs1137101 variant was observed at a significance level of 0.05. Similarly, a deviation from the Hardy-Weinberg equilibrium was observed in the patient group with the *LEP* rs7799039 variant. (TABLE 2) We considered that the deviation in the patient group for the *LEP* rs7799039 variant may be due to selection favoring heterozygosity. However, the deviation observed in the *LEPR* rs1137101 variant in the control group may be attributed to genetic drift and gene flow, possibly resulting from the geographical context of Turkey, which regularly receives migration from various populations. The relationship between the *LEP* rs7799039 and *LEPR* rs1137101 genotype distribution and demographic and clinical characteristics, such as sex, fasting blood glucose, hypertension, triglyceride, retinopathy, nephropathy, neuropathy, coronary heart disease, cerebrovascular accident, BMI, waist circumference, HbA1C, insulin, C-peptide, creatinine, blood urea nitrogen, uric acid, AST, ALT, LDL, HDL, total cholesterol, triglyceride, Hb, and spot urine protein/creatinine ratio, were also analyzed. For *LEP* rs7799039, those with the AA-AG genotypes had a higher mean triglyceride level (*P* = .032) than those with the other genotypes. For *LEPR* rs1137101, both AA and AA-GA genotypes were higher in females than in males. Furthermore, the genotype combination of AA-GA was observed to be more prevalent in females than the AA genotype.( *P* = .045). Moreover, patients with the AG-GG genotype had a lower mean Hb level (*P* = .018) than those with the AA genotype. The relationship between *LEP* rs7799039 and *LEPR* rs1137101 genotype distribution and clinical characteristics is shown in TABLES 3 and 4.

**TABLE 3.** Relation of *LEP* rs7799039 Genotype Distribution with Clinical Findings

	*LEP* rs7799039		OR Exp (B)	95% CI	*P*
	GA-AA n = 116 (%)	GG n = 34 (%)			
Gender					
Female, n (%)	68 (58.62)	24 (70.58)	0.590^b^	0.259-1.347^b^	.235^b^
Male, n (%)	49 (42.24)	10 (29.42)			
Fasting blood glucose>100 mg/dL or DM	115 (99)	34 (100)	0.991^b^	0.975-1.008^b^	.000^b^
Hypertension	93 (80)	30 (88)	1.420^a^	0.437-4.611^a^	.560^a^
Female-HDL^c^	40 (35)	16 (47)	1.643^a^	0.600-4.503^a^	.334^a^
Male-HDL^c^	33 (28)	7 (21)	1.080^a^	0.242-4.815^a^	.919^a^
Triglyceride^d^	76 (66)	16 (47)	0.504^a^	0.230-1.107^a^	.088^a^
Retinopathy (+)	10 (9)	4 (12)	1.191^a^	0.330-4.291^a^	.790^a^
Nephropathy (+)	15 (13)	4 (12)	0.930^a^	0.280-3.095^a^	.906^a^
Neuropathy(+)	10 (9)	5 (15)	2.006^a^	0.607-6.628^a^	.254^a^
CHD (+)	15 (18)	8 (24)	1.831^a^	0.656-5.108^a^	.248^a^
CVA (+)	1 (1)	0 (0)	1.399^b^	0.227-8.624^b^	1.000^b^
^a^OR (95%CI) was adjusted by age and sex, ^b^Fisher’s Exact Test, ^c^Normal values for HDL for females >50 mg/dL, for males > 40 mg/dL, ^d^Upper level of normal for triglycerides is 150 mg/dL.					
	Mean (min-max)	Mean (min-max)			
BMI (kg/m^2^)	31 (23-51)	32 (24-52)			.249^a^
Waist circumference (cm)	102 (75-170)	102 (90-129)			.804^a^
Fasting blood glucose (mg/dL)	121 (84-525)	124 (1-378)			.710^a^
HbA1c	6.4 (4.7-12.2)	6.65 (4.7-11.6)			.329^a^
Insulin (mIU/mL)	11.7 (2.3-81)	11.8 (3.5-49)			.864^a^
C-peptide (ng/mL)	3 (1.6-10.5)	3 (0.5-8)			1.000^a^
Creatinine (mg/dL)	0.76 (0.4-7.9)	0.8 (0.5-1.36)			.559^a^
BUN (mg/dL)	14 (5-38)	13.6 (9-52)			.880^a^
Uric acid (mg/dL)	4.9 (2.9-11.4)	4.8 (2.9-11.4)			.690^a^
AST (U/L)	17 (8-80)	17 (9-36)			.896^a^
ALT (U/L)	17 (5-138)	17 (9-42)			.339^a^
Total cholesterol (mg/dL)	194 (112-311)	195 (143-305)			.881^a^
LDL (mg/dL)	123 (37-198)	117 (79-234)			.845^a^
HDL (mg/dL)	44 (26-75)	45 (28-74)			.976^a^
Triglyceride (mg/dL)	179 (48-707)	146 (53-335)			**.032** ^a^
CRP (mg/L)	3.4 (0.2-35)	3.2 (0.3-62)			.845^a^
Hb (g/dL)	13.1 (10.3-17)	12.6 (9.6-17)			.506^a^
Spot urine protein/creatinine	0.87 (0.49-4.6)	0.1 (0.05-0.9)			.830^a^

a Independent-samples median test.

ALT, alanine transaminase; AST, aspartate transaminase; BUN, blood urea nitrogen; CHD, coronary heart disease; CRP, C-reactive protein; CVA, cerebrovascular accident; DM, diabetes mellitus; Hb, hemoglobin; HDL, high-density lipoprotein; HT, hypertension; LDL, low-density lipoprotein

**TABLE 4.** Relation of *LEPR* rs1137101 Genotype Distribution with Clinical Findings

	*LEPR* rs1137101		OR Exp (B)	95% CI	*P*
	AA n = 72 (%)	AG-GG n = 78 (%)			
Gender					
Female, n (%)	38 (57.77)	54 (69.24)	0.497^a^	0.255-0.968^a^	**.045** ^**a**^
Male, n (%)	34 (47.23)	24 (30.76)			
Blood glucose >100 mg/dL or DM	71 (99)	78 (100)	0.986^a^	0.959-1.014^a^	.480^a^
Hypertension	62 (86)	61 (78)	0.451*	0.178-1.141*	.092*
Female-HDL^b^	24 (33)	32 (41)	0.811*	0.341-1.930*	.635*
Male-HDL^b^	22 (31)	18 (23)	1.672*	0.519-5.389*	.390*
Triglyceride (mg/dL)^c^	40 (56)	52 (67)	1.706*	0.864-3.366*	.124*
Retinopathy (+)	6 (8)	8 (10)	1.450*	0.448-4.699*	.535*
Nephropathy (+)	7 (9)	12 (15)	1.924*	0.689-5.368*	.211*
Neuropathy (+)	10 (14)	5 (6)	0.479*	0.152-1.508*	.208*
CHD (+)	13 (18)	10 (13)	0.702*	0.272-1.812*	.464*
CVA (+)	1 (1)	0 (0)	1.399^a^	0.227-8.624^a^	1.000^a^
*OR (95%CI) was adjusted by age and sex; ^a^Fisher’s exact test; ^b^Normal values for HDL for females is greater than 50 mg/dL, for males is greater than 40 mg/dL; ^c^Upper level of normal for triglycerides is 150 mg/dL					
BMI (kg/m^2^)	31 (23-51)	31 (23-46)			.860^d^
Waist circumference (cm)	102 (78-170)	102 (72-525)			.974^d^
Fasting blood glucose (mg/dL)	122 (1-378)	120 (1-378)			.633^d^
HbA1c (%)	6.5 (5-13)	6.4 (4.7-11.6)			1.000^d^
Insulin (mIU/mL)	12 (5-66)	11 (2.3-81)			.214^d^
C-peptide (ng/mL)	3 (1.3-5.87)	2.9 (0.5-10.5)			1.000^d^
Creatinine (mg/dL)	0.8 (0.48-1.9)	0.75 (0.4-1.36)			.414^d^
BUN (mg/dL)	14 (5-52)	13 (6-36)			.161^d^
Uric acid (mg/dL)	4.9 (2.9-11.4)	4.9 (2.4-12.5)			.655^d^
AST (U/L)	18 (8-72)	17 (9-80)			.257^d^
ALT (U/L)	19 (8-138)	18 (5-105)			.688^d^
Total cholesterol (mg/dL)	185 (112-311)	199 (114-281)			.453^d^
LDL (mg/dL)	120 (37-234)	123 (61-214)			1.000^d^
HDL (mg/dL)	42 (26-75)	50 (28-75)			.113^d^
Triglyceride (mg/dL)	169 (48-707)	174 (65-585)			.742^d^
CRP (mg/L)	3.1 (0.2-62)	3.7 (0.3-55)			1.000^d^
Hb (g/dL)	13.4 (10.7-16.8)	12.3 (9.6-17)			**.018** ^d^
Spot urine protein/creatinine	0.83 (0.05-1.6)	0.1 (0.05-4.6)			.310^d^

d Independent-samples median test.

ALT, alanine transaminase; AST, aspartate transaminase; BUN, blood urea nitrogen; CHD, coronary heart disease; CRP, C-reactive protein; CVA, cerebrovascular accident; DM, diabetes mellitus; Hb, hemoglobin; HDL, high-density lipoprotein; HT, hypertension; LDL, low-density lipoprotein

## Discussion

MetS is typically characterized by the accumulation of central adiposity, which is known to cause a range of metabolic disorders that increase the risk of developing cardiovascular diseases and diabetes. These metabolic disorders include dyslipidemia, hypertension, and insulin resistance, which are commonly associated with MetS.^13^ The pathophysiology of MetS involves several complex mechanisms that have not been fully elucidated. Incorporating genetic and epigenetic elements and lifestyle and environmental factors, such as excessive consumption and insufficient physical activity, also make a significant contribution to the development of this syndrome. Visceral adipose tissue (VAT) is fat that accumulates around organs in the abdominal cavity. Excessive VAT intake is strongly associated with MetS.^14^ Individuals with excess VAT often have higher LEP levels. Although LEP levels may be elevated, the brain can potentially become desensitized to its impact. This condition is known as LEP resistance and is a common feature of MetS. Nesrine et al^15^ reported that circulating LEP levels were correlated with body adiposity in adults and children. It is assumed that high LEP levels in obese individuals are evidence of LEP resistance. In ob/ob mice, although there was no change in the weight of the mice, glucose and insulin levels decreased in the hours after LEP replacement,^16^ whereas the administration of LEP antagonists increased blood glucose and insulin levels before changes in body weight.^17^ Intravenous injections of LEP have been shown to normalize hyperglycemia and hyperinsulinemia and improve insulin sensitivity in lean mice.^18^

That *LEP* gene mutations and defects in leptin receptors cause extreme hyperphagia and obesity is already known.^19^ According to certain research, variations in the *LEP* and *LEPR* genes have been linked to the underlying mechanisms of obesity and diabetes. The *LEP* rs7799039 variant in the promoter of the gene is hypothesized to affect transcription levels and LEP expression.^20^ The A allele of *LEP* rs7799039 was associated with twice as much leptin secretion from adipocytes as the G allele.^21^ As the GA genotype of *LEP* rs7799039 is associated with hyperglycemia and hypercholesterolemia, it is mostly associated with the development of MetS. *LEPR* rs1137101 in exon 6, which is putatively the LEP-binding site, impairs leptin-binding activity.^22^

Studies have investigated the association of *LEP* rs7799039 and *LEPR* rs1137101 polymorphisms with obesity, diabetes, insulin resistance, dyslipidemia, and cancer.^23-25^ Dagdan et al^26^ showed that the A allele of *LEP* rs7799039 increased serum LEP levels among Mongolians. In addition, this polymorphism was associated with elevated BMI and fasting glucose levels. A study by Mirrakhimo et al^27^ revealed that the *LEPR* rs1137101 polymorphism was associated with higher insulin resistance in the Kyrgyz population. Shramko et al^28^ found that the A allele of the *LEP* rs7799039 gene was the most frequent in MetS patients. In the same study, the highest systolic blood pressure was associated with the rs7799039 GG genotype. Bains et al^29^ reported that *LEP* rs7799039 and *LEPR* rs1137101 are associated with T2DM in the North Indian Punjabi population. In a meta-analysis examining 5143 T2DM cases and 5021 controls, *LEPR* rs1137101 was shown to be associated with T2DM in all genetic models.^30^ In a study from our country, Turkey, the GA and AA genotypes of *LEP* rs7799039 were shown to be predictors of increased BMI in obese patients.^31^ Furthermore, it was determined in another study that the co-occurrence of *LEP/LEPR* GG/GG genotypes elevated the risk of obesity among Turkish patients.^32^ Moreover, research that included a pediatric group revealed that *LEPR* gene polymorphisms were not related to obesity or metabolic syndrome in Turkish children.^33^ Baumaiza et al^31^ reported that *LEP* rs7799039 and *LEPR* rs1137101 polymorphisms and haplotype combinations were related to MetS and obesity in the Tunisian population. However, there are also studies showing that there is no relationship between *LEP* rs7799039 polymorphism and obesity.^34,35^ There are conflicting results regarding the relationship between the *LEPR* gene and obesity.^36,37^

This study evaluated the relationship between *LEP* rs7799039 and *LEPR* rs1137101 and MetS risk and MetS parameters in the Turkish population. In this single-center study, we obtained demographic and clinical information on the individuals. A novel insight of this study is that the GA and AA genotypes and the A allele of *LEP* rs7799039 were associated with MetS. This was in line with many studies examining the relationship between the *LEP* rs7799039 polymorphism and MetS, T2DM, and obesity. Patients with the G allele of *LEPR* rs1137101 have an increased risk of MetS. We also evaluated the relationship between the genotype distribution of these variants and demographic and clinical findings. Those with GA-AA genotypes had higher triglyceride levels. The AG and GG genotypes of *LEPR* rs1137101 were higher in females than in males.

Our study has some limitations. First, although the number of participants was relatively large, the sample size of certain subgroups was limited and insufficient. As MetS is a multifactorial disease, it is difficult to determine the effect of heredity on the disease. Moreover, we focused on only 2 SNVs in the LEP and LEPR pathways; other functional SNVs should be considered in future studies. However, an advantage is that our study sample consists of people who are close to each other in terms of age and gender and represent Turkish society.

## Conclusion

MetS is a complex disease that poses a worldwide threat and incurs high socioeconomic costs. As MetS is a risk factor for many diseases, it should be carefully evaluated. Determining the risk factors for MetS will be beneficial for the prevention and treatment of this disease. In summary, our findings suggest that *LEP* rs7799039 and *LEPR* rs1137101 are associated with the risk of MetS development and metabolic syndrome–related disorders in the Turkish population.

## Data Availability

All data and materials supporting the results or analyses presented in this study are available upon reasonable request.

## Abbreviations

MetS: metabolic syndrome
LEP: leptin
LEPR: LEP receptor
T2DM: type 2 diabetes
HDL: high-density lipoprotein
SNVs: single nucleotide variants
BMI: body mass index
HbA1c: hemoglobin A1c
AST: aspartate transaminase
ALT: alanine transaminase
LDL: low-density lipoprotein
CRP: C-reactive protein
OR: odds ratio
VAT: visceral adipose tissue

## Notes

This study was approved by the Research Ethics Committee of Istanbul University (approval No. 2022/1310) and was conducted in accordance with the Declaration of Helsinki.

Author Contributions: Conceptualization: M.J., N.S., M.K.; Data curation: M.J., M.D.G.; Formal analysis: F.C.T., Y.O.; Investigation: F.C.T., Y.O.; Methodology: M.J., N.S., M.K.; Project administration: M.G.D., S.P.; Resources: N.S., F.C.T.; Supervision: S.P., A.M.; Validation: M.J., S.P., A.M.; Writing-original draft: M.J., N.S., M.G.D.; Writing-review and editing: S.P., A.M
